# Infliximab Precision Dosing in Inflammatory Bowel Disease (IBD) Patients: A Review of Current Literature

**DOI:** 10.7759/cureus.76424

**Published:** 2024-12-26

**Authors:** Alexandros Toskas, Magdalini Manti, Nikolaos Kamperidis, Naila Arebi

**Affiliations:** 1 Gastroenterology, St Mark's Hospital and Academic Institute, London, GBR

**Keywords:** bayesian models, inflammatory bowel disease, infliximab, precision dosing, ulcerative colitis (uc)

## Abstract

The therapeutic failure of infliximab therapy remains a challenge in patients with inflammatory bowel disease (IBD), and dose optimization is often required. Accelerated or intensified regimes showed value in treating patients in the acute setting with high CRP or low albumin levels, which are suggested by recent guidelines; however, evidence is weak. Therapeutic drug monitoring (TDM), with anti-tumor necrosis factor-alpha (TNF-α) trough levels and antibodies, showed value during maintenance therapy, but not in induction and can guide clinical decisions in patients that might be undertreated with the standard dosing regimen. Combining the impact of therapeutic drug monitoring with a Bayesian forecasting methodology to calculate drug clearance can help on calculating the optimal infliximab dose for patients with ulcerative colitis (UC) and Crohn’s disease (CD) on both the induction and maintenance phase. This will help to identify those who need intensification of their current regime to boost the therapeutic effect and those who are non-responders. This review aims to summarize the recent literature regarding infliximab precision dosing in IBD patients using forecasting methodology.

## Introduction and background

The therapeutic options for inflammatory bowel disease have significantly expanded in the last decade, with novel classes of biologics and small molecules emerging in the market. However, infliximab (IFX), a chimeric monoclonal antibody against tumor necrosis factor-alpha (TNF-α), remains the drug of choice in acute severe ulcerative colitis (UC) and the preferable first-line choice in biologic naïve patients with moderate to severe UC or Crohn's disease (CD) [1]. Recent data have also suggested a top-down approach in newly diagnosed active Crohn's disease with better outcomes in one year, supporting early use of infliximab with thiopurines [2]. In the pediatric population, anti-tumor necrosis factor-alpha (TNF-α) remains the first-line therapy for children (six to 17 years old) with moderate to severe IBD [3]. Therefore, maximizing the effectiveness of anti-TNF treatment is a top priority.

Despite the high clinical response rate during induction, standard dosing (three-dose induction regimen at weeks zero, two, and six, followed by maintenance therapy every eight weeks) was associated with the risk of losing response that is reported in a recent study as high as 37% for CD patients. The risk of loss of response (LOR) was calculated at 13% per patient-year [4]. Previous studies had shown interindividual variability for infliximab clearance with several factors that can lower drug exposure, including body weight, age <10 years old, low serum albumin, extensive disease, neutrophil CD64, Oncostatin M (OSM), OSM receptor (OSMR) [5,6]. The current strategy of drug monitoring, with drug trough levels being measured right before the upcoming biologic infusion, may lead to a delayed change in implementation and dose optimization. By utilizing the trough and antibody levels to create precision pharmacokinetic models, the response and durability of infliximab can increase, and the clinician will receive guidance on the appropriate initial dosing to estimate future trough concentrations [3]. Bayesian models are based on established pharmacokinetic models. Still, they combine additional information by therapeutic drug monitoring (TDM) levels and patient factors with minimized entry errors since Bayesian systems focus less on values that seem to deviate significantly from prior information [7].

Our review aims to summarize the existing studies on using precision pharmacokinetic models to calculate the infliximab dosage and guide induction and maintenance regimens.

## Review

Materials and methods

**Figure 1:**
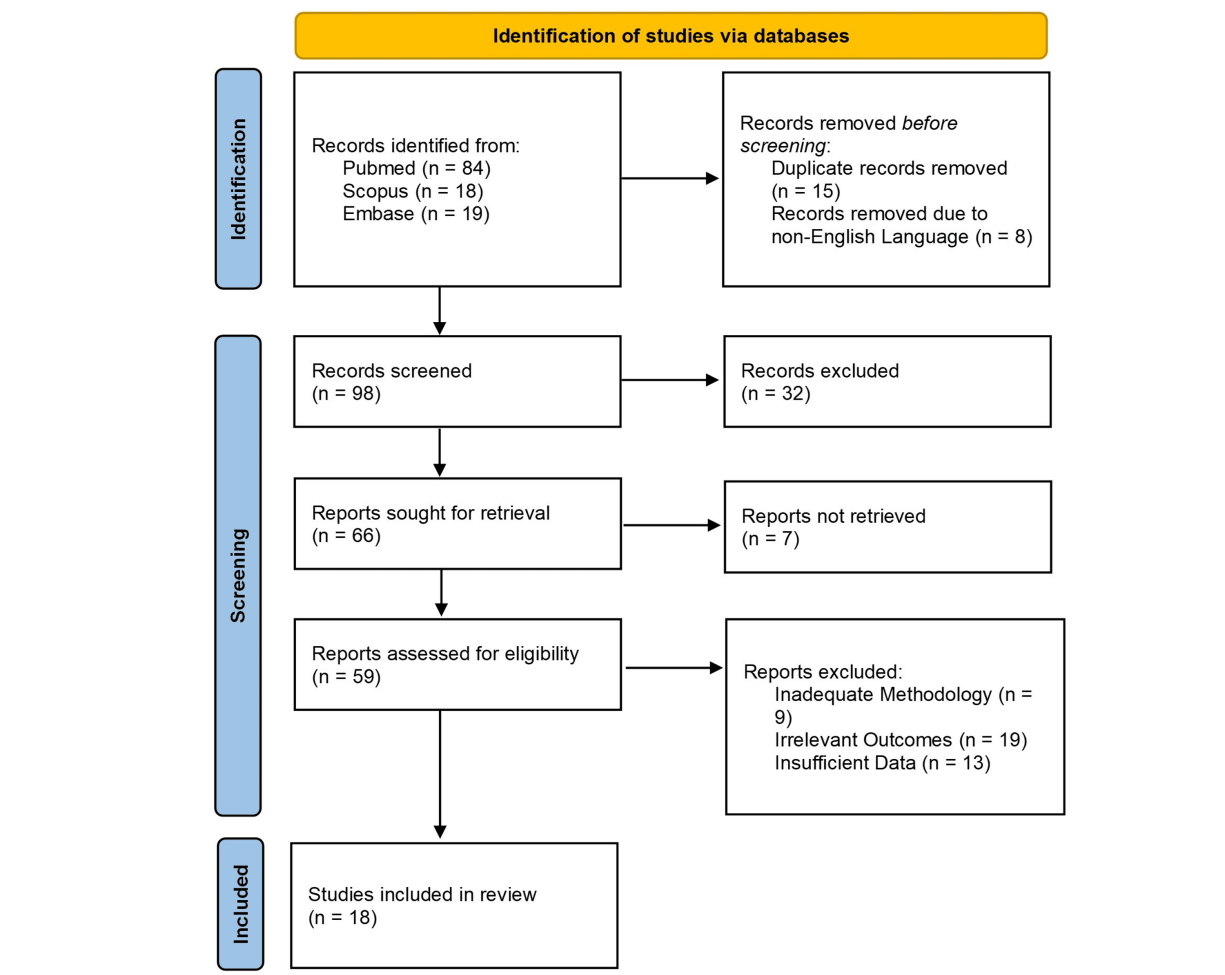
PRISMA flow diagram illustrating the methodology of literature search. PRISMA: Preferred Reporting Items for Systematic Reviews and Meta-Analysis.

Three databases (Pubmed, Scopus, and Embase) were searched using the terms ‘bayesian,' ‘IBD,' 'infliximab dose optimization,' and 'model.' References were checked between 2014 and 2024. In total, 121 results were searched and analyzed as per Figure 1 following the Preferred Reporting Items for Systematic Reviews and Meta-Analysis (PRISMA) guidelines. Studies using empirical infliximab dosing, inadequate methodology, insufficient data, or irrelevant outcomes were excluded. In total, 18 results were analyzed. Eight studies used IFX precision dosing models, as shown in Table 1.

**Table 1:** Summary of studies regarding IFX precision dosing in IBD. IFX: infliximab, IBD: inflammatory bowel disease, UC: ulcerative colitis, LOR: loss of response, PK: pharmacokinetic, MIPD: model-informed precision dosing, QI: quality improvement, CD: Crohn's disease.

Number	Authors	Year	Population	Results
1	Laurence et al. [8]	2022	140	Optimizing induction with proactive trough levels resulted in higher post-induction levels and greater odds of attaining long-term clinical remission.
2	Strik et al. (precision study) [9]	2021	80 (66 CD, 14 UC)	Reduced LOR in UC patients in remission treated with precision, algorithm based, dosing.
3	Abraham et al. [10]	2023	275	69% of patients with IBD treated with IFX were predicted to benefit from dose optimization during the induction phase of therapy and at week 52.
4	Dave et al. [7]	2020	30	73% concurrence between predicted and observed IFX levels with iDOSE. Individualized IFX dosing and duration are feasible.
5	Bossoyt et al. [11]	2021	187	Ultraproactive drug monitoring had equal results with reactive drug monitoring in maintenance IBD patients.
6	Buurman et al. [12]	2015	42 (8 UC/34 CD)	PK model based on trough levels showed that dosing can be reduced to 12 weekly.
7	Kantasiripitak et al. [13]	2023	31	Deep endoscopic remission at six months in children using MIPD.
8	Piester et al. [14]	2018	49	The median IFX trough levels of >5 μg/mL were achieved with 81% probability, increased by 12% compared with pre-QI values.

Discussion

Therapeutic Drug Monitoring

Infliximab trough levels have been the standard of care in calculating IFX doses after induction [15]. However, they lack accuracy. Their value was underlined in previous studies in both adult and pediatric populations. Lawrence et al. [8] included 140 children with IBD in an international retrospective cohort study. One group received a standard induction regime with three doses at weeks zero, two, and six. The second group used the dose optimization strategy based on clinical response, biomarkers (CRP), and IFX trough levels. The IFX dose could be increased to 10 mg/kg, or the infusion intervals could be shortened to a minimum of every four weeks. TDM was also measured in the second group before the fourth dose of IFX. The researchers concluded that steroid-free remission was higher in the dose-escalation group at week 52 when compared to the standard group (65/78 (83%) versus 32/62 (52%), P<0.001). Moreover, both the biomarker remission (69/78 (88%) versus 44/62 (71%), P=0.009) and the combined steroid-clinical and biomarker remission (65/78 (83%) versus 25/62 (40%), P<0.001) were higher in the optimized group. In terms of immunomodulator usage, fewer patients in the optimized group were treated with azathioprine or 6-mercaptopurine compared to the standard group (p<0.001). In fact, an observational study indicated that proactive TDM in IFX monotherapy was equally effective when compared to IFX-immunomodulator combination therapy [16]. More cost-effective biomarkers and clinical factors are to be introduced for dose optimization. In another retrospective study by Hofmekler’s team, 129 children with IBD from Atlanta were studied. Researchers showed that decreasing the infusion intervals from eight to six weeks during maintenance was more efficient when compared to dose escalation (p=0.009 versus p=0.674) in terms of achieving the optimal IFX levels. The main principle was that low IFX levels lead to higher percentages of anti-infliximab inhibitory antibodies (ATI) (50% of patients with IFX levels below 3 μg/mL). Furthermore, when TDM was implemented, a high percentage of patients with IFX levels below 3 μg/mL was identified; thus, either early TDM or expedition of the third dose before the sixth week should be adopted in the daily practice [17]. Still, this study mainly included patients diagnosed with CD; only 29 patients had UC. Although joint studies imply that IFX doses are equivalent in both entities, further studies are needed to verify these findings.

Point of care testing (POCT) for infliximab levels and antibodies is a quick diagnostic method that can be performed in the outpatient setting instead of the classic enzyme-linked immunosorbent assay (ELISA) method. Apart from prediction models, these strategies were also suggested as part of IFX dose monitoring, but not always successfully [18]. In a trial published by Bossuyt et al., an ultra-proactive strategy, instead of the known proactive one, was compared to the standard reactive approach of trough levels. The primary outcome was the percentage of IFX failure in one year, while secondary outcomes were the number of trough-level tests, the percentage of interval modifications, and the number of patients with clinical remission. One hundred eighty-seven patients in the phase of IBD maintenance were divided into two distinct groups: the ultra-proactive and the reactive group. In the first group, trough IFX levels were measured via ELISA in all patients at the beginning of the trial. The desired range was 3-7 μg/ml. If IFX levels were above that range, an interval prolongation was suggested. In the case of lower-level detection, though, the infusion interval was reduced by two weeks, and trough levels were measured on a POCT. However, the outcomes exhibited that there was no statistically significant difference after one year in terms of IFX failure between the two groups (19% versus 10%; p=0.08). The secondary outcomes similarly showed no difference in clinical remission (75% versus 83%, p=0.17). Mucosal healing, though, was surprisingly higher in the second group (p=0.021) [11]. The latter outcome entails selection bias since only 38% of the patients had undergone an endoscopy.

Bayesian Forecasting and Model-Informed Precision Dosing

During the clinical management of IBD, weight-based models are mostly used to calculate the IFX dose. The dose optimization strategies so far are based on empirical guidance lacking accuracy. However, there is variability in pharmacokinetics (PK), and most precision models support the use of Bayesian software (BS) and model-informed precision dosing (MIPD) to optimize the infliximab dosing. This software calculates the medication clearance and monitors it over the course of treatment. The BS uses population PK models as priors to achieve this. Even in asymptomatic patients, clearance monitoring could predict forthcoming disease flares and the development of ATI [19]. 

On the multicenter 1:1 trial, both UC and CD patients in remission were randomized either in guided IFX dosing or conventional treatment. On this cohort of 80 patients, 88% from the guided dosing group were in clinical remission one year after, versus 64% of the conventional treatment group (P<0.017). Patients in the precision dosing group had lower calprotectin levels as well. This was one of the first studies that have demonstrated a reduced incidence of loss of response (LOR) to IFX [9].

In the PRECISION trial, Abraham et al. have provided clinicians with a precision-guiding dosing test for patients with IBD on maintenance (PredictPK IFX tool) [10]. This precision-guided dosing tool combined logistic regression and machine learning, based on Bayesian population pharmacokinetic models and was able to forecast IFX trough levels. Two hundred seventy-five patients attended the study. Treatment modification included not only an increase in dose but also a de-escalation. No ATIs were detected when the dose de-escalation was chosen. The study showed that 81% of patients have improved with dose optimization, and 58% of health practitioners have modified their management plan based on this [10].

While the PRECISION trial and its subsequent tools were validated in the Western population, iDose software attempted to investigate the response of Asian patients. Once again, based on a Bayesian algorithm, the iDose dashboard system aimed to predict the IFX concentration and, consequently, suggest dose adjustments for non-responders. The information added to the system was the following: current IFX dose, disease severity based on IBD scores, CRP, albumin and previous IFX levels or ATI if available. Thirty patients with IBD took part in the study, and dose modification was implemented for 11 of them based on iDose calculations. Among this subpopulation, one patient did not respond to dose adjustment and was found to have already developed ATI. Overall, iDose was beneficial, especially in Asian predictive models, given the percentage of NUDT15 variants, up to 10.7%, among this population, which increases the risk of thiopurine toxicity. Thus, accurate prediction of IFX levels is of major importance. iDose correctly predicted IFX levels in 21 out of 30 patients; the remaining nine had inadequate information, and their laboratory tests (CRP, albumin) were changed throughout the disease progression [7]. Still, 30 patients are a relatively small population, and more patients need to be recruited in observational studies to verify these predictive tools.

On the other hand, Buurman et al. developed a PK model based on trough levels and have shown that 12-week dosing can be considered in patients who had not developed anti-infliximab inhibitory antibodies (ATI) and had reduced IFX therapy-related hospital visits by one-third. Albumin, gender, and level of ATIs were shown to be statistically significant covariates in drug clearance in this study [12]. Similarly, Lega et al. used IFX concentration levels and ATI to guide the dosing and timing of the first maintenance infusion, with IFX level being both dose- and frequency-adjusted when IFX levels were <20 ug/mL [16].

In a pediatric study, Kantasiripitak et al. evaluated eight pediatric PK models and created a MIPD software tool to predict IFX levels. The primary outcome was a week 12 infliximab concentration target of 7.5 mg/L, which was associated with a 64% probability of deep remission at six months. They studied a group of 31 children that received 5 mg/kg IFX as an induction dose. The week 12 target was achieved with maximum precision and accuracy when they used the week six infliximab concentration levels, and a significant difference was found compared with standard dosing patients. This model has improved the deep remission rates in children with IBD. However, since this was a pediatric population with a weight below 40 kg, dose calculation may be flawed, and it is one of the reasons for non-responders [13].

Piester et al., in their prospective study, developed a web-based IFX dosing calculator using the known variables of CRP, albumin, body weight, disease activity index, and calprotectin through IFX and ATI levels. A mixed population of 49 pediatric and adult IBD patients was studied for nine months, and maintenance dosing was received based on this calculator in a hospital-based infusion unit. An IFX trough level >5 μg/mL was achieved with a probability of 81%, which was a significant increase compared with conventional dosing [14].

## Conclusions

Optimizing IFX dosing for IBD patients remains a crucial aspect of treatment due to the challenge of inadequate dosing. This can lead to unnecessary treatment modifications, delaying clinical effectiveness and affecting patients' quality of life. In addition, traditional empirical approaches result in delayed adjustments and potential undertreatment, highlighting the need for more proactive strategies. TDM had shown promise in maintaining therapy but is underutilized during induction, leading to potential undertreatment. Combining TDM with Bayesian forecasting methods offers a promising avenue for calculating optimal IFX doses, considering individual patient factors and pharmacokinetics. Recent literature has explored various approaches, including clearance monitoring, ensuring adequate trough levels, and the development of predictive tools such as the PredictrPK IFX tool and iDose software. In the pediatric population, initiatives like the MIPD and mobile IFX dosing calculator (mIDC) have shown efficacy in optimizing doses. However, challenges remain, including the economic burden of TDM and the need for larger studies to validate predictive models. Overall, a shift of the current practice towards more personalized and proactive dosing strategies would be promising for the improvement of outcomes in IBD patients receiving IFX therapy.
